# Measures against Plague

**Published:** 1901-08-17

**Authors:** 


### MEASURES AGAINST PLAGUE.

Our whole conception of the subject of plague, and especially of its prevention, is wonderfully simplified if we will but regard it as primarily an epizootic disease communicable to man rather than an epidemic disease communicable to animals. Dr. Davies, medical officer of health for Bristol, urges that it is high time that we should adapt our measures for the exclusion of the disease to the newly ascertained conditions instead of going on blindly along routine lines which, although sanctioned by tradition and custom, are quite inapplicable to the end in view. We may well ask, with Dr. Davies, whether the measures which our port medical officers are at present empowered to adopt for the exclusion of plague are most accurately adapted to its special nature and etiology, for in the words of Professor Koch, "it is a great blunder to treat pestilences uniformly." The present Local Government

Board regulations against cholera, yellow fever, and plague, which are all grouped together, were issued in 1896 and are practically a transcript of former regulations against cholera, and no further official instructions relating to plague were issued until the spring of this year, when a memorandum was issued by the Local Government Board giving sanction to certain measures which the local sanitary authorities had found it necessary to take in regard to ships carrying infected rats. Our knowledge as to the etiology of plague has, however, arrived at a point when we are certainly justified in asking, as Dr. Davies now asks, whether it is enough merely to put in force the powers of the sanitary authorities against ships known or suspected to have on board infected rats, and whether the time has not come when we should demand that all merchant ships should be rendered rat-free. It is impossible to regard with satisfaction a position which allows droves of infected animals to be carried about from port to port and leaves the protection of the towns to the chance of such success as may happen to attend the efforts made to prevent the landing of these rats. "To bring infected rats to a port in unlimited numbers and then rely on attempts to prevent them coming on shore is obviously attacking the evil at the wrong end. It is impossible to destroy all rats on a loaded vessel; it is not a very difficult matter to practically secure this on an empty vessel." Clearly the policy to aim at is to secure rat-free merchant ships; and to show that this is possible Dr. Davies quotes the experience of Australia, where, as the result of inter-colonial agreement, unless vessels can produce a certificate of fumigation, they are liable to detention. Under these conditions it is found that if vessels which engage in coasting voyages lasting three weeks to a month are fumigated at the port of departure before loading and at the ultimate port touched on the voyage, few and often no rats are discovered on their return. This statement, he says, applies to steam vessels up to 4,000 tons. Undoubtedly it must be regarded as a mistake to go on at great expenditure of time and trouble enforcing certain red-tape measures for the inspection of the passengers, for the detention of "contacts," for taking the addresses of all on board, and for communicating with the sanitary officers of the districts to which they are proceeding, when really it is not a question of passengers but of rats.

